# Feasibility and diagnostic added value of a point of care mobile EEG system

**DOI:** 10.1016/j.cnp.2026.07.014

**Published:** 2026-07-24

**Authors:** Sidsel Armand Larsen, Louise Klok, Louise Maria Andersen, Marlene Linnebjerg Kudsk, Pirgit Meritam Larsen, Anne Sabers, Sándor Beniczky

**Affiliations:** a Danish Epilepsy Centre, Filadelfia, Denmark; b University of Copenhagen, Denmark; c Brain Capture, Lyngby, Denmark; d The Citizen Centre for People with Disabilities, Copenhagen, Denmark; e The Epilepsy Clinic, Copenhagen University Hospital Rigshospitalet, Denmark; f Department of Clinical Neurophysiology, Copenhagen University Hospital Rigshospitalet, Denmark

**Keywords:** Point-of-care EEG, Decentralized diagnostics, Intellectual disability, Epilepsy, Residential care facilities, Non-expert EEG acquisition

## Abstract

**Objective:**

EEG is an essential diagnostic tool in epilepsy. Many patients with intellectual disability and epilepsy reside in residential care facilities and do not have access to EEG, as the modality is not available on-site. Additionally, due to poor cooperation, transporting these patients to specialized hospital-based EEG centers is often difficult or impossible resulting in lack or delayed diagnostic evaluation. Mobile, point-of-care EEG systems, with electrodes that can be easily applied outside the hospital by caregivers without formal training in EEG recording, have the potential to bridge this diagnostic gap.

**Methods:**

In this prospective multicenter study, we assessed the feasibility and clinical utility of a point-of-care EEG system in residential care facilities. EEG electrodes were applied by local personnel without prior training in EEG recording. Data was uploaded to a secure cloud platform and remotely interpreted by experts.

**Results:**

We recruited 25 participants. EEG recording could be completed in 22 participants. The recordings were clinically interpretable in 17 participants and provided complete answers to the referrals in 11 participants. No technical failures of the EEG device or data transmission were observed.

**Conclusions:**

Our results demonstrate the feasibility and clinical utility of point-of-care mobile EEG for the diagnosis of epilepsy in residential care facilities.

**Significance:**

Point-of-care mobile EEG has the potential to improve equity in access to neurophysiological diagnostics.

## Highlights

• Point-of-care EEG was completed in 22 of 25 included residential-care patients.
• Recordings were clinically interpretable in 77% of the completed studies.
• EEG answered the referral question in 65% of the interpretable recordings.

## 1 Introduction

Electroencephalography (EEG) is an essential diagnostic modality in the evaluation of patients presenting with seizures or suspected epilepsy and is typically performed in specialized hospital-based EEG centers (Benbadis et al., 2020; Tatum et al., 2018). The prevalence of epilepsy among individuals with intellectual disabilities is substantially higher than in the general population (Robertson et al., 2015), resulting in a considerable need for EEG assessments in this group. However, patients with intellectual disability and epilepsy often experience difficulties related to hospital-based EEG monitoring. Many of these patients live in residential care facilities and transportation to hospital-based EEG facilities is often logistically challenging and, in many cases, not feasible due to behavioral difficulties, or poor cooperation. As a result, clinically indicated EEG examinations may be delayed or not performed at all, contributing to diagnostic uncertainty, misdiagnosis and suboptimal seizure management, in this vulnerable patient population.

Implementing EEG in an out-of-hospital setting has the potential to improve equity in access to neurophysiological diagnostics and enhance the likelihood of obtaining clinically interpretable data (Milne-Ives et al., 2023). Decentralized acquisition models, in which EEG is recorded locally and interpreted remotely by specialists, may further improve the quality of care for this patient group while minimizing disruption to their daily lives. However, evidence regarding the real-world feasibility and diagnostic contribution of point-of-care EEG systems operated by non-expert personnel in residential care settings remains limited. A previous study on healthy volunteers has shown that a point-of-care EEG system is suitable for recording clinically relevant EEG recordings, even when operated by inexperienced users (Armand Larsen et al., 2024). Therefore, a portable point-of-care EEG system designed for simplified electrode application by non-expert personnel represents a potential strategy to address this diagnostic gap.

In this prospective multicenter study, we evaluated the technical feasibility and diagnostic added value of a point-of-care mobile EEG system implemented in residential care facilities for patients with intellectual disability and epilepsy. We hypothesized that this point-of-care mobile EEG system used by non-expert personnel would yield clinically interpretable routine EEG recordings and contribute to diagnostic clarification in a clinically meaningful proportion of cases.

## 2 Material and methods

**Fig. 1:**
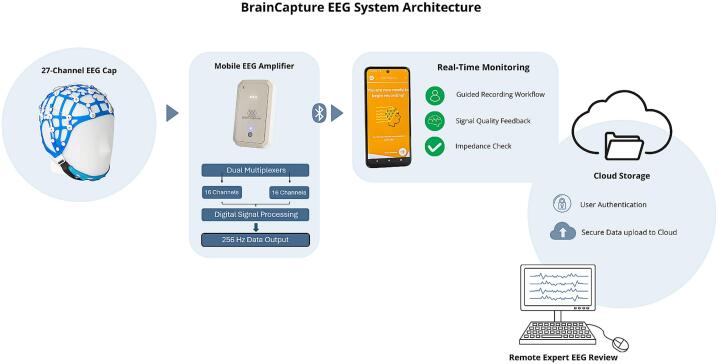
BrainCapture EEG system architecture. A 27-channel EEG cap is connected to the mobile EEG amplifier, processing recorded data. The amplifier is connected to a mobile application via Bluetooth. The mobile application provides real-time guided monitoring with signal quality feedback allowing the inexperienced user to correct for inadequate signal quality. The recorded EEG is stored securely in the cloud, permitting remote expert EEG review.

**Fig. 2:**
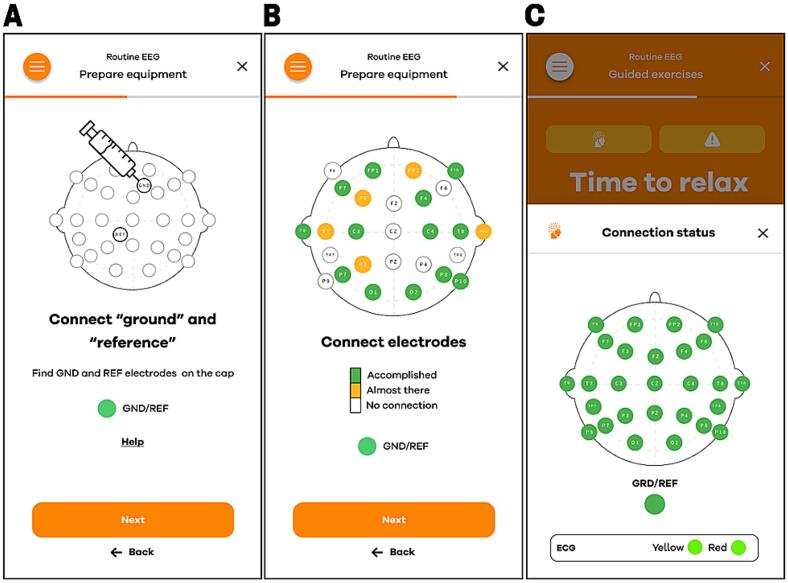
Real-time quality control in the mobile application. Screenshots from the mobile application show how the real-time quality control is performed in the guided workflow. A and B showing the connectivity during gel-up of the cap allowing the user to follow progress. C showing impedance check during the recording allowing the user to correct if signal quality is inadequate.

We evaluated a mobile, point-of-care EEG system - BrainCapture EEG device (BC-1), which comprises a 27-channel EEG amplifier that wirelessly transmits data via Bluetooth to a mobile application. The system architecture (Fig. 1) is based on sequential channel sampling, utilizing two multiplexers that each process 16 channels. The signals undergo digital processing and reconstruction to the desired 256 Hz high-quality data sampling rate for each channel. In the mobile application, a real-time quality control algorithm is incorporated that continuously monitors impedance and signal quality, and gives immediate feedback if electrodes are disconnected or the signal quality is inadequate (Fig. 2). Recording workflow is managed by the mobile application that guides the inexperienced user to perform an EEG recording and afterwards securely uploads the data to a cloud-based storage platform. Stored recordings were accessed by user authentication, enabling remote expert review. We used BrainCapture BC-Caps with embedded Ag/Cl electrodes for EEG recordings and for connectivity between scalp and cap Weaver Nuprep skin prep gel and Sanismart conducting gel were used (Armand Larsen et al., 2024).

The project was approved by the Institutional Review board (EMN-2024-04445) and written informed consent from a caregiver was obtained before an EEG recording was performed.

The project was conducted at residential and day-care facilities affiliated with the Danish Epilepsy Center (Stormly, Juelsminde and Brommeparken, Munke Bjergby), and at residential care facilities within the Municipality of Copenhagen. Eligible participants were residents with intellectual disability living in the participating residential care facilities who had a clinical indication for a routine EEG recording. Indications for referral included: (1) acute clinical suspicion of seizure activity or status epilepticus, or (2) other clinical question for which EEG evaluation was considered necessary by the clinician in charge (e.g., ictal EEG or participants who had never previously undergone EEG examination due to poor cooperation). Residents meeting these criteria were prospectively referred to an EEG recording performed on site at their residential care facility. Residents who were unable or unwilling to cooperate with the EEG recording were excluded. Intellectual disability was assessed according to the ICD-11 classification (WHO, 2025).

Four nurses employed at the residential care facilities received a brief training in EEG recording (Armand Larsen et al., 2024) with the BC-1 system. The nurses had no prior experience or knowledge of recording an EEG. The training consisted of a one-day training course in operating the app, placing the cap, and gel-up the cap. After a short training, the nurses independently performed EEG recordings. Following referral for EEG, the trained nurse coordinated and performed the recording at the residential care facility, thereby enabling the patient to remain in a familiar and comfortable environment.

Two independent EEG experts evaluated the recordings remotely. In cases of disagreement between the two readers, a third reader was consulted to achieve majority consensus. The primary outcomes were technical quality of the recordings, the proportion of clinically interpretable recordings, and the impact on clinical decision-making. A recording was considered clinically interpretable if the reviewing EEG expert assessed that the EEG recording included sufficient good-quality EEG data to make a reliable clinical interpretation.

As a secondary outcome, patient experience and cooperation were assessed by questionnaires and rated on a 1–5 scale. Furthermore, we used the Danish translation of the System Usability Scale (SUS-DK) (Hvidt et al., 2020) to measure the perceived usability of the staff who performed the EEG recordings. The SUS score was calculated using the method developed by John Brooke (Brooke, 1996).

The primary endpoint was the feasibility of the recording using the portable device, defined as the proportion of patients in whom the EEG recording can be successfully completed. We expected a feasibility of 95%. With a 10% margin of error, a sample size of 19 patients was calculated using the standard formula for estimating a single binomial proportion (Cochran, 1977). Generative artificial intelligence tools were used to assist with language editing, structural suggestions and improvement of clarity. All content was critically reviewed and revised by the authors, who take the full responsibility for the final manuscript.

## 3 Results

Twenty-five participants with intellectual disability living in a residential care facility and fulfilling the inclusion criteria were recruited to the study (August 2024–February 2026). The study population consisted of 16 males and 9 females, with a median age of 37 years (range 11–74 years). Of the 25 participants, three had moderate intellectual disability, one had severe intellectual disability and 21 had profound intellectual disability. Recordings were performed at the residential care facility by nursing staff well-known by the patient. One patient was referred due to acute clinical suspicion of seizure activity, six for ictal EEG recording and 15 for assessment of interictal epileptiform discharges.

EEG recordings were successfully completed in 22 of 25 recruited participants (88%). EEG recordings could not be completed in three participants (two males and one female) due to insufficient cooperation during the procedure. The three participants comprised one individual with moderate intellectual disability and two with profound intellectual disability. Prior to the EEG examination, the staff had familiarized the participants with the EEG cap through practice sessions. Despite these efforts, these participants were unable to complete the recordings.

No technical failures of the EEG device or data transmission were observed during the study period.

Clinically interpretable EEG recordings were obtained in 17 of 22 recordings (77%). Median recording length was 30:19 min (IQR: 00:30:10–00:46:29), with durations ranging from 00:10:30 to 02:06:38. In five cases, the EEG recordings were not interpretable because the participants could not cooperate resulting in short recording duration or excessive muscle and movement artifacts dominating the recording. The five participants with non-interpretable EEG recordings, all had profound intellectual disability. Reduced cooperation was observed in these participants and was characterized by difficulty remaining still during the EEG recording, leading to frequent movement artifacts. In addition, some participants attempted to remove the EEG cap, interrupting the recording process and compromising the EEG quality.

In 11 of the 17 interpretable recordings (65%) the EEG findings provided sufficient information to address the clinical question posed by the referring physician. The remaining six recordings were of acceptable signal quality but failed to answer the clinical question.

The visually inspected EEG recordings demonstrated abnormal findings in 13 of the 17 clinically interpretable recordings, while four recordings were considered normal. Background abnormalities were observed in ten recordings, diffuse slowing in seven recordings and focal slowing in two recordings. Epileptiform activity was identified in ten recordings, including generalized epileptiform discharges in three recordings and focal or multifocal epileptiform discharges in seven recordings. One recording showed an ictal EEG pattern. Some recordings exhibited more than one of these features. Overall, the EEG findings reflected that the study cohort included different EEG features ranging from background slowing to focal or generalized epileptiform activity.

Furthermore, all participants were reported to have a positive experience during the EEG recording in the residential care home. Caregivers considered that the key factors contributing to a positive experience were the opportunity to remain in an environment well-known by the participants and to receive reassurance from familiar nursing staff. Examples included the possibility for participants to undergo the recording while resting in their own bed, holding hands with known caregivers, going out in the garden during recording or engaging with familiar toys or sensory objects. These examples were perceived as important in facilitating cooperation with the EEG recording procedure.

**Fig. 3:**
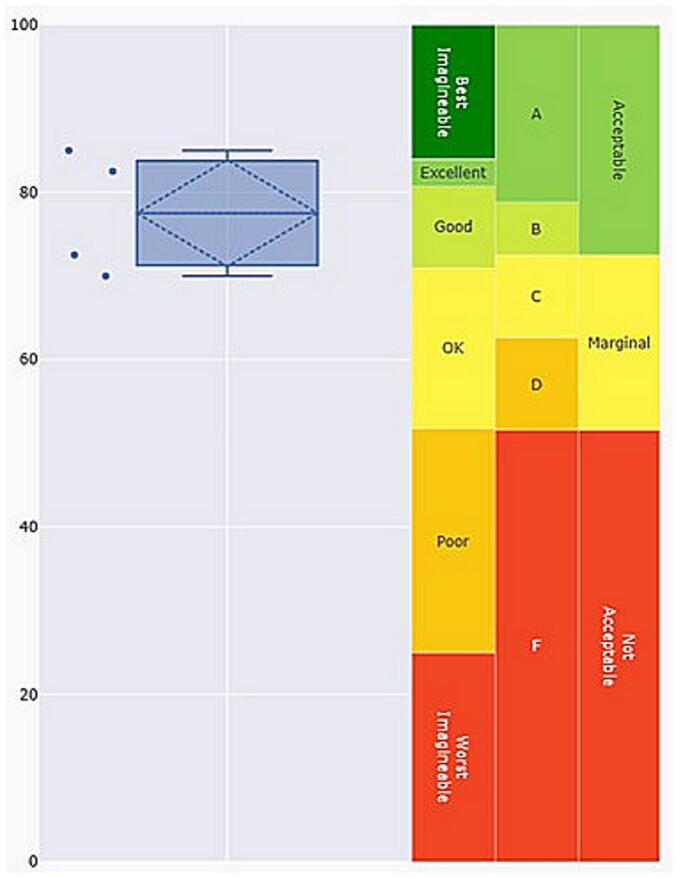
System Usability Scale (SUS) scores and corresponding qualitative interpretation Left panel illustrating boxplot for the distribution of SUS scores obtained from the users of the BC-1 device. The central line indicates the median, the box the interquartile range and whiskers the range. On the side are individual data points represented.

All four users (i.e. personnel who did the recordings) rated the BC-1 system on the System Usability Scale resulting in an average score of 78 (range from 67.5 to 90) on a 0–100 scale (Fig. 3). User experience varied considerably, with individuals having conducted between 1 and 20 EEG recordings. Overall, users reported high satisfaction with the device and the users agreed that the functions, as real-time guidance and continuous feedback, were well integrated in the system. In addition, the users were asked to describe aspects of the device they found difficult to use, challenges encountered while using the device and how the device could potentially be improved. The primary challenge reported by the users was the gel-up process, which was considered difficult and time-consuming, particularly in this patient population.

The right side illustrates a contextualization scale mapping numerical scores to qualitative descriptors, adjective ratings, acceptability range and corresponding grade classifications and with a visual color gradient ranging from red to green indicating a transition from unacceptable to highly acceptable usability. A mean SUS score of 78 is interpreted as ‘good’ on the adjective rating scale and lies within the acceptable usability range. The figure was created using a web-based analysis toolkit for the system usability score (Blattgerste et al., 2022).

## 4 Discussion

In this prospective, multicenter study, we evaluated the feasibility and clinical utility of a point-of-care mobile EEG system implemented in residential care facilities for individuals with intellectual disability with epilepsy or suspicion of epilepsy. EEG acquisition was not feasible in all referred individuals, as expected given the practical challenges associated with recording EEG in individuals with intellectual disability. Non-interpretable EEG recordings were observed in participants with severe or profound intellectual disability, which likely reflects the greater challenges associated with performing EEG examinations in individuals with more severe cognitive impairment. In this context, the successful completion of EEG recordings in most referred participants and the high proportion of clinically interpretable recordings support the feasibility of the approach. Importantly, the EEG findings contributed to answering the referring clinician's question in half of the recordings, which is similar to the diagnostic yield of EEGs recorded in specialized centers. These findings suggest that decentralized EEG acquisition in residential care facilities is feasible and can provide clinically meaningful diagnostic information in a patient population that often faces substantial barriers to conventional hospital-based EEG examinations.

Technological development in portable and rapid EEG systems have facilitated the decentralization of neurophysiological examinations including emergency departments, intensive care units and patient's homes (Herman, 2026; Patedakis Litvinov et al., 2026). A key innovation is the development of portable, easy-to-use systems that can be deployed quickly by nonspecialists personnel (Patedakis Litvinov et al., 2026). Point-of-care EEG systems may be particularly valuable for diagnosing acute neurological emergencies such as nonconvulsive status epilepticus and nonconvulsive seizures, enabling faster seizure detection and timely treatment (Herman, 2026). Several previous studies have evaluated point-of-care EEG systems across a range of clinical settings and consistently demonstrated their feasibility when deployed outside specialized neurophysiology units. In pediatric and neurological emergency departments, simplified EEG applied by non-EEG specialists enabled rapid achievement of clinically useful recordings that supported seizure detection and treatment decisions, and functioning as a supplement to routine clinical assessment (Simma et al., 2025; Welte et al., 2024).

In addition, previous studies have evaluated and validated the implementation of various other decentralized EEG modalities, including home video-EEG monitoring for long-term video-EEG monitoring (Milne-Ives et al., 2023). This approach has primarily been investigated in adult individuals. Overall, the available evidence suggests that decentralized EEG acquisition can yield clinically useful information comparable to conventional hospital-based EEG examinations. However, only a limited number of studies have included individuals with intellectual disability (Brunnhuber et al., 2014; Kandler et al., 2017) The existing data nevertheless indicates that decentralized EEG monitoring is feasible in this patient group and may improve equity in access to neurophysiological diagnostics (Milne-Ives et al., 2023). This is in concordance with our study where we were able to perform clinically and technically relevant recordings in most of the participants with intellectual disabilities.

Moreover, the experience with decentralized EEG recordings for both patients and the caregivers has been reported as favorable and are generally preferred over hospital admissions (Kandler et al., 2017). This was preferred due to increased comfort in undergoing the procedure in a familiar home environment with support from known staff. Similar observations were made in the present study, where caregivers highlighted the benefits of performing EEG recordings in the residential care home, including avoidance of stressful transportation and exposure to unfamiliar hospital environments. In our study we succeeded with decentralized EEG acquisition in some participants who have never undergone conventional EEG before, with caregivers arguing that a comfortable environment and well-known staff as the key. Furthermore, previous studies suggested that decentralized EEG examinations may reduce healthcare costs compared to hospital-based examinations (Brunnhuber et al., 2014; Green et al., 2023)*.* Although cost-effectiveness was not evaluated in the present study, this may represent an additional potential benefit and a potential area for future evaluation. Evidence from feasibility and implementation studies have also demonstrated strong clinical interest in integrating point-of-care EEG systems into clinical practice, especially for monitoring drug-resistant epilepsy, detecting nocturnal seizures, and potentially reducing seizure-related mortality risks (Biondi et al., 2024)*,* which is often seen in this patient population. However, implementation of a point-of-care mobile EEG system in residential care facilities for individuals with intellectual disability has received limited attention.

These findings extend previous work by demonstrating that decentralized EEG acquisition is feasible – not only in acute hospital environments, or for long-term monitoring in patients home supported by experts, but also in residential care homes for individuals with intellectual disabilities supported by non-expert users. A wider implementation of such solutions may contribute to more equitable access to epilepsy care and timely identification of clinically relevant information. Therefore, future research should focus on confirming diagnostic yield, evaluating cost-effectiveness, and determining the impact of using decentralized EEG acquisition on a larger and more diverse population.

The users in this study reported a high SUS score for the BC-1 system with a score of 78, which is generally interpreted as indicating good usability and acceptable system performance (Fig. 3). This score exceeds the commonly reported acceptability threshold of approximately 68 (Sauro, 2018) suggesting that the system can be operated effectively by users with limited prior EEG experience. The usability assessment should be interpreted in light of the small number of respondents completing the System Usability Scale. However, these findings support the feasibility of deploying the system in decentralized clinical settings. Furthermore, high usability represents an important criterion for successful implementation. By enabling EEG acquisition outside specialized hospital-based centers, such systems may contribute to improve equity in access to neurophysiological diagnostics. In addition, users identified the gel-up process as the most challenging part of using the BC-1 device, as it was considered time-consuming and particularly difficult in this patient population because limited patient cooperation often complicates EEG preparation. This did not appear to substantially affect the overall usability, as reflected by the high SUS scores. Further optimization of the BC-1 device by reducing the preparation time could be done by reducing the number of electrodes in the cap, thereby shortening the electrode preparation time while still maintaining sufficient clinical information. Furthermore, alternative electrode technologies, such as dry electrodes or pre-gelled electrodes, may further simplify EEG preparation and improve usability. Although these approaches were beyond the scope of the present study, they warrant consideration in future development of the device.

The main limitation of this study is the small sample size of users, which should be considered when interpreting the findings, as it limits the generalizability of the study. However, the primary objective of this study was to evaluate feasibility of the BC-1 system, defined as the proportion of patients in whom the EEG recording can be successfully completed. In addition, the BC-1 system does not enable simultaneous video recording, and therefore no contextual clinical information was available during EEG interpretation. Our study does not include a comparator group undergoing conventional hospital-based EEG and therefore cannot establish diagnostic equivalence between the two approaches. However, this was not the objective of the study. The portable point-of-care EEG was used in patients for whom conventional in-hospital EEG was not feasible - consequently, no direct comparator recordings were available.

### 4.1 Conclusion

In conclusion, point-of-care mobile EEG acquisition in residential care facilities is feasible and can provide clinically relevant information in a substantial proportion of individuals with intellectual disability and epilepsy. Decentralized EEG systems may represent a promising approach to improving access to neurophysiological diagnostics in medically complex patient populations.

## Funding

This study received no external funding.

## Declaration of competing interest

The authors declare that they have no known competing financial interests or personal relationships that could have appeared to influence the work reported in this paper.
